# Lizards as sentinels for the distribution of *Angiostrongylus cantonensis*

**DOI:** 10.1017/S0950268824000931

**Published:** 2024-12-13

**Authors:** Lucia Anettová, Vojtech Baláž, Radovan Coufal, Michal Horsák, Elena Izquierdo-Rodriguez, Anna Šipková, Pilar Foronda, David Modrý

**Affiliations:** 1 Department of Botany and Zoology, Faculty of Science, Masaryk University, Czech Republic; 2 Department of Ecology and Diseases of Zoo Animals, Game, Fish and Bees, Faculty of Veterinary Hygiene and Ecology, University of Veterinary Sciences Brno, Brno, Czech Republic; 3 Department of Zoology, Faculty of Science, Palacký University Olomouc, Czech Republic; 4 Institute of Parasitology, Biology Center of Czech Academy of Sciences, Czech Republic; 5 Instituto Universitario de Enfermedades Tropicales y Salud Pública de Canarias, Universidad de La Laguna, La Laguna, Canary Islands, Spain; 6 Department of Obstetrics and Gynecology, Pediatrics, Preventive Medicine and Public Health, Toxicology, Legal and Forensic Medicine and Parasitology, Universidad de La Laguna, La Laguna, Canary Islands, Spain; 7 Department of Veterinary Sciences, Faculty of Agrobiology, Food and Natural Resources/CINeZ, Czech University of Life Sciences Prague, Czech Republic

**Keywords:** *Angiostrongylus cantonensis*, caudal autotomy, lizards, rat lungworm, sentinels

## Abstract

The rat lungworm *Angiostrongylus cantonensis* is a zoonotic metastrongyloid nematode currently considered an emerging pathogen. Originating in Southeast Asia, this nematode has spread to tropical and subtropical parts of the world via its invasive rodent and gastropod hosts.

On the island of Tenerife in the Canary archipelago, the *A. cantonensis* invasion was recognized more than a decade ago. The endemic lizard *Gallotia galloti* has been identified as a paratenic host of this nematode in the Canary Island ecosystem. Because this lizard species is the most abundant reptile in Tenerife, we tested its suitability as a possible sentinel for *A. cantonensis* presence. Lizards were captured alive in nine localities, spanning an environmental gradient across the island. Tail muscle tissue was obtained by provoked caudal autotomy and tested for the nematode infection by a species-specific qPCR. Infection intensities were assessed by detecting *A. cantonensis* DNA quantities based on a calibrated standard curve. Of the 129 samples tested, 31 were positive. The prevalence varied among localities, with the highest (63.6%) recorded in a humid laurel forest. Even though the prevalence in Valle San Lorenzo was the lowest, this is the first record of *A. cantonensis* from the arid south of Tenerife. Variation in prevalence at different localities was significantly and positively correlated with increasing vegetation cover and negatively correlated with seasonal variability of precipitation, as determined by Spearman correlation coefficients. Fisher’s exact test was used to determine the variation in the prevalence of *A. cantonensis* among adult males, females, and juveniles and showed no significant difference. Also, there was no significant difference in infection intensity between males and females (as determined by GEE-g). We demonstrated that provoking caudal autotomy can be an effective non-lethal method of *A. cantonensis* mapping in island ecosystems with abundant lizard species, particularly those with a sharp climatic and vegetation gradient, from xeric to humid conditions.

## Key results

- *Angiostrongylus cantonensis*, an invasive zoonotic nematode causing meningitis in humans, is present throughout the island of Tenerife.
- Abundant lizards are suitable sentinels of the parasite’s occurrence as they are present in all habitats.
- Provoked caudal autotomy provides an effective, non-lethal method of sample collection.

## Introduction

Reptiles are known to be involved as paratenic hosts in the life cycle of *A. cantonensis* (Metastrongyloidea: Angiostrongylidae), a zoonotic nematode responsible for human neuroangiostrongyliasis, commonly known as rat lungworm disease and presenting as eosinophilic meningitis. [1–4]. In addition to affecting humans, *A. cantonensis* can cause neurological disorders in other mammalian and avian aberrant hosts. The parasite, also called the rat lungworm, typically cycles between various species of rats as definitive hosts and a range of terrestrial and aquatic gastropods as intermediate hosts [5].

*A. cantonensis* is presumed to have originated in Southeast Asia, and its continuing spread, associated with rodent and gastropod hosts, is a prominent example of biological invasion. It has been spreading throughout many tropical and subtropical regions, and global awareness of it is increasing among researchers and public health officials [6]. In the past decade, the emergence of infection foci in Europe has attracted considerable attention. The first possible evidence of *A. cantonensis* in Europe was an autochthonous case of human eosinophilic meningitis reported in France, though the source of infection was unknown [7]. The parasite was later found in hedgehogs in Mallorca [8] and it has recently been reported in rats in mainland Spain [9]. The Canary Islands represent a well-known hotspot of the parasite’s distribution close to continental Europe and within the formal area of the European Union. The rat lungworm was discovered in rats in Tenerife in 2010 [10] and is well established in the humid northern part of the island [11].

The distribution of *A. cantonensis* is normally monitored via detection in its definitive or intermediate hosts [12–14], sometimes complemented by case reports of autochthonous human or animal infections (i.e., in aberrant hosts). Less commonly, its presence is reported in paratenic hosts such as amphibians and reptiles [15]. Recently, the lizard *Gallotia galloti* (Sauria: Lacertidae) was reported as a paratenic host of *A. cantonensis* in Tenerife [3, 16]. This omnivorous terrestrial lizard, endemic to Tenerife and La Palma, is an extreme habitat generalist, occurring in all types of terrestrial habitats from coastal areas up to 3,000 m above sea level [17]. Several factors make it a promising sentinel for monitoring the parasite’s distribution: it is abundant throughout the island, it is relatively easy to catch, and it was classified as Least Concern with a stable population by IUCN [18].

Spontaneous caudal autotomy is well-known in a range of lizards as a defensive strategy for last-minute escape from predation [19]. This is associated with a constriction of blood vessels to reduce blood loss in predetermined fracture planes, which in most lizard species are present in each of the posterior caudal vertebrae, with the exception of the few closest to the cloaca [20]. This phenomenon incurs costs for the individual, such as energetic loss, impairment of reproduction, and a decline in social status [19]. Tissue from tails released during spontaneous caudal autotomy can be used to screen for the presence of the parasite. The AcanR3990 qPCR assay, as demonstrated in our prior study, efficiently detects *A. cantonensis* DNA in both liver and tail muscles [3], making it a valuable tool for targeted surveillance in various hosts with a detection limit of 10^−5^ larvae per sample [21].

In this study, we surveyed *G. galloti* from various habitats in Tenerife to evaluate overall *A. cantonensis* infection prevalence and intensity in this paratenic host across different habitats. We also assessed whether the stage of maturity or sex of lizards had any impact on prevalence and intensity. Additionally, we highlight the potential of adopting a non-lethal approach, i.e., replacing lizard sacrifice with collection of tissue released during provoked caudal autotomy.

## Materials and methods

### Study area and localities

**Figure 1.**
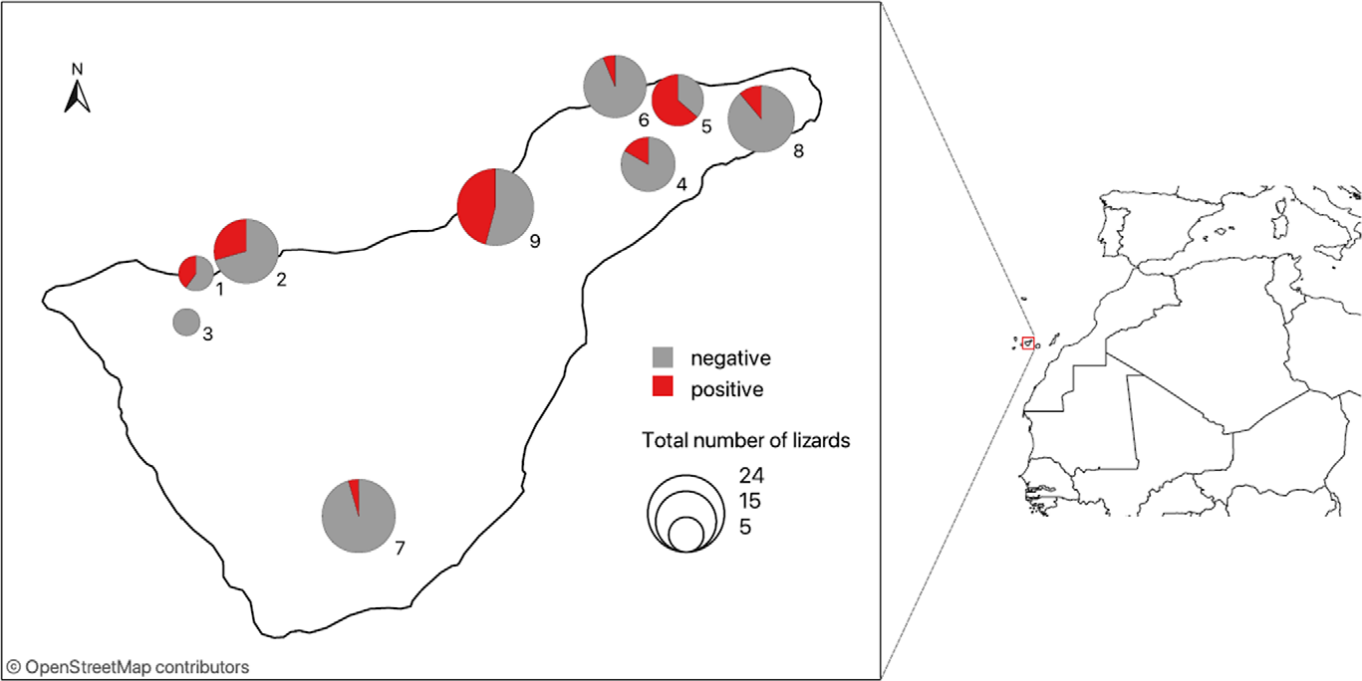
Localities on Tenerife from which lizards *Gallotia galloti* were screened for the presence of *Angiostrongylus cantonensis*, with the prevalence of the parasite’s DNA in tail tissue depicted in pie charts. Numbers of localities corresponding to Table 1 are stated next to the pie charts.

Tenerife is a volcanic island in the centre of the Canary archipelago with a warm, dry climate. Its diverse ecosystems range from xeric shrub habitats at lower elevations to thermophile and laurel forests in higher northern areas. Nine localities, each covering an area of approximately 200 m^2^, were selected for sampling of *G. galloti* throughout the island (Figure 1) spanning a gradient of vegetation cover and biomass, categorized from low (1) to high (5): (1) bare rocks with only succulents (Valle San Lorenzo, Tejina, Maria Jimenez), (2) sparse bush (Garachico), (3) continuous bush with no trees (Tierra del Trigo/Tanque, La Orotava/El Rincon), (4) continuous bush with isolated trees (Erjos and Tegueste), and (5) woodland (Anaga). This range of localities covers most of the climatic variability of the island.

**Table 1.** Localities for sampling of *Gallotia galloti* for the detection of *Angiostrongylus cantonensis* across Tenerife, Canary Islands. Erjos was excluded from the statistical analysis

Locality		Coordinates	No. of collected individuals	No. of positive individuals	Percentage of positive individuals	Vegetation type	Elevation (meters)	Annual temperature (°C)	Annual precipitation (mm)	Precipitation seasonality (%)
Loc 1	Tierra del Trigo/Tanque	28.359145 N, 16.789473 W	5	2	40	3 - continuous bush	460	17.18	332.00	78.08
Loc 2	Garachico	28.369805 N, 16.760223 W	17	5	29.4	2 - sparse bush	160	19.19	271.00	80.56
Loc 3	Erjos	28.315665 N, 16.789395 W	3	0	0	4 - bush with isolated trees	1,180			
Loc 4	Tegueste	28.525941 N, 16.339430 W	11	4	36.4	4 - bush with isolated trees	370	17.25	365.00	78.77
Loc 5	Anaga	28.531214 N, 16.308671 W	11	7	63.6	5 - woodland	660	16.02	414.00	76.75
Loc 6	Tejina	28.535917 N, 16.356945 W	16	1	6.3	1 - bare rocks with only succulents	260	18.63	312.00	80.93
Loc 7	Valle San Lorenzo	28.091800 N, 16.650066 W	22	1	4.6	1 - bare rocks with only succulents	420	18.57	303.00	79.71
Loc 8	Maria Jimenez	28.504412 N, 16.230386 W	17	2	11.1	1 - bare rocks with only succulents	70	19.87	288.00	81.33
Loc 9	La Orotava (El Rincón)	28.410708 N, 16.512897 W	24	13	54.2	3 - continuous bush	190	19.36	283.00	78.66

Climatic data (mean annual temperature, annual precipitation, and precipitation seasonality) were extracted from the WorldClim v1.4 database [22] for each study locality using its geographic coordinates and the ArcGIS 8.3 program [23]. Precipitation seasonality is a standard deviation of the monthly precipitation estimates expressed as a percentage of the mean of those estimates (i.e. the annual mean). This is a measure of the variation in monthly precipitation totals over the course of the year, expressed as a percentage, with larger percentages representing greater variability of precipitation.

### Lizard sampling

Lizards were captured alive with traps (5-l plastic jars baited with a ripe tomato and into which the lizard would fall and be unable to climb out), which were set during the day next to rock walls (for approximately 8 h) and checked every hour in April – May 2021 and 2022. We collected 129 individuals, including seven found as road-killed specimens. We examined the tail muscle tissues of all seven road-kill lizards, and in five of them, we were also able to analyze liver tissue. All lizards were categorized as juveniles and adults (according to their size). Adults were further identified as males or females, based on their typical colour features [24], assessed solely by the naked eye to minimize handling stress. Juveniles were not sexed because of the absence of fully developed colour features. After capturing the lizards, their tails were grasped to induce caudal autotomy, regenerated parts of tails were not included in the analyses. The separated part of the tail, about two-thirds of its total length, was retained as a sample before releasing the lizard. Approximately 25 mg of tissue was extracted from the most proximal part of the obtained tail muscle and was kept in a freezer (−18 °C) until DNA extraction. As a reserve, the rest of the tail was also stored in a freezer.

### DNA extraction and qPCR analysis for *A. cantonensis*

Muscle tissue samples from the tails were defrosted and weighed. Approximately 25 mg of tissue (proximal part of tail) was cut into small pieces and used for DNA extraction with a DNeasy Blood&Tissue (Qiagen, Germany) extraction kit with modifications optimized for infectious third-stage larvae (L3) of *A. cantonensis*, with the pre-lyse phase extended overnight (instead of 10 min). All the tail muscle (*n* = 129) and liver (*n* = 5) samples were examined for the presence of *A. cantonensis* DNA by a species-specific qPCR assay [20]. The assay was performed in a 20 μL reaction using 6.2 μL of PCR water, 10 μL of 2× MasterMix (IDT Prime time gene expression master), 0.2 μL of 10 μM probe (PrimeTime Eco Probe 5′ 6-FAM/ZEN/3′ IBFQ, /56-FAM/ACA TGA AAC/ZEN/ACC TCA AAT GTG CTTCGA/3IABkFQ/), 0.8 μL of each 10 μM primer (forward: AAA CTG TTG CTT TCG AAG CTA TG and reverse: GCG CAA ATC TGA CGT TCT TG) and 2 μL of DNA template. Thermocycling (40 cycles) was conducted as follows: 95 °C for 20 s followed by 40 °C for 1 s and 60 °C for 20 s. DNA from a single third-stage larva of *A. cantonensis* extracted by the same method as the samples and diluted 100× was used as a positive control. Nuclease-free water was used as a negative control. The assay was run in duplicates. The Ct value (average value between duplicates) of positive samples was calculated by absolute quantification analysis of the 2nd Derivative Maximum [25]. Only amplification curves with a Ct value under 35 were considered positive so as to avoid false positive results due to amplification and fluorescence artifacts, or cross-contamination. Quantification of larvae per gram of processed tissue sample was performed by using a standard curve (Supplementary Figure S1), which was calculated using serial dilutions (1×, 10×, 100× and 1000×) of DNA extracted from a single L3. This standard curve was then applied for all qPCR runs, with a positive control of 100× dilution used as a calibrator. The amount of *A. cantonensis* DNA per sample was subsequently recalculated for the actual weight of the sample used during extraction.

### Statistical analyses

To associate the percentage of positive lizard individuals at each locality with vegetation structure, elevation, and main climatic parameters (i.e., mean annual temperature, annual precipitation, and precipitation seasonality), the Spearman correlation coefficient was used. Fisher’s exact test was used to test the difference in frequency of positive and negative individuals among females, males, and juveniles. Generalized estimating equations with a Gaussian error structure (GEE-g) and location as a random factor were used to examine the differences in infection intensity between positive females and positive males. Juveniles were not considered as only one positive juvenile was recorded. Infection intensity values were ln-transformed prior to modelling to obtain a normal distribution. Locality 3 (Erjos) was excluded from all calculations as only three individuals were collected, which is not sufficient for a reliable assessment of the nematode infection level. All analyses and graphics were performed and generated in R software 4.1.0 [26] using the vegan [27] and geepack [28] packages.

## Results

In total, 122 live-trapped and seven road-killed *G. galloti* individuals were collected from nine localities across Tenerife (Figure 1). Of 129 samples of tail tissue, 35 tested positive for *A. cantonensis* DNA by qPCR. In the five specimens for which both tail and liver tissues were examined, one lizard tested positive in the tail and the liver, while the remaining four lizards were negative in both tissue samples. The lizards tested positive for *A. cantonensis* in localities of all vegetation types, although the prevalence varied (among localities as well as among vegetation types) (Table 1, Figure 1). We collected 38 males, of which 13 tested positive, and 81 females, of which 20 tested positive. Of the 10 juveniles collected, two tested positive. Based on Fisher’s exact test, there were no significant differences (*p* = 0.4955) among males, females, and juveniles in the frequency of positive and negative individuals. There was also no significant difference in infection intensity between females and males (GEE-g, *p* = 0.4996). The results of the qPCR quantification (amount of L3 DNA) for comparing infection intensities are listed in Supplementary Table S1.

**Figure 2.**
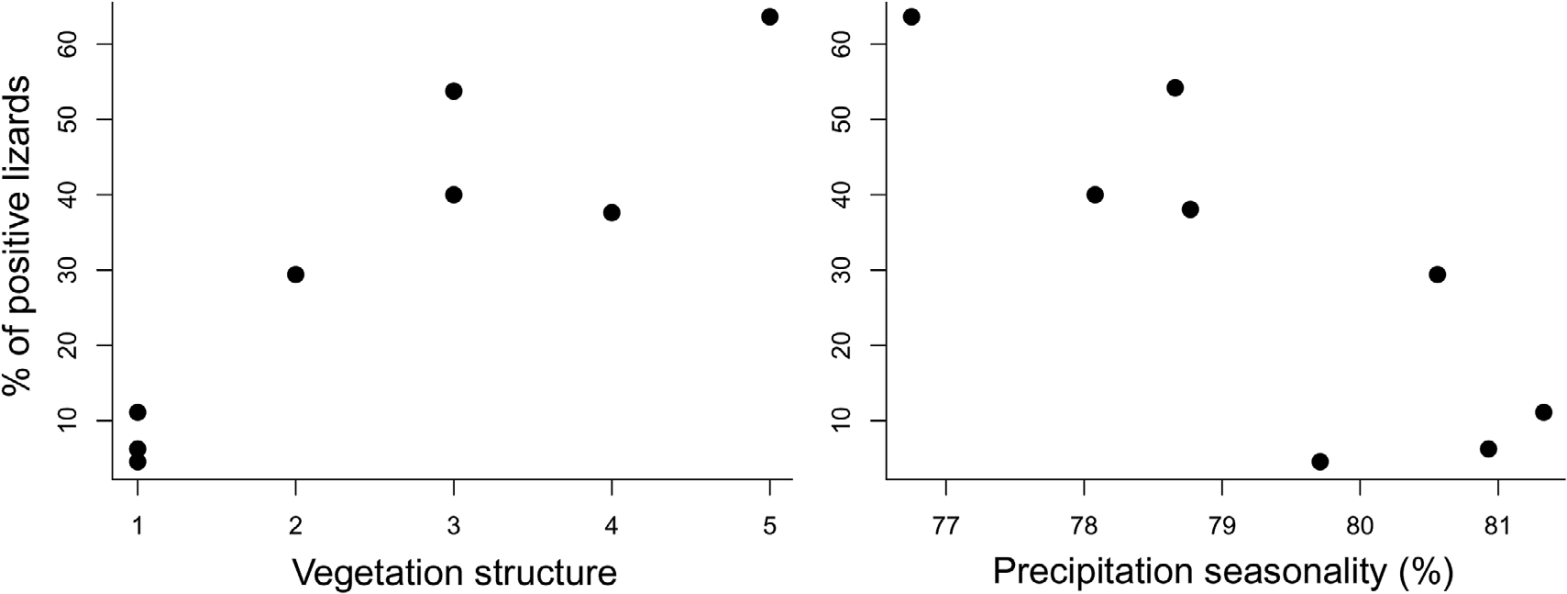
Relationship between percentage of positive lizard individuals (y) and vegetation structure or precipitation seasonality (x). Each dot represents a sampling locality.

Variation in prevalence at localities was significantly and positively correlated with vegetation structure. It was also significantly negatively correlated with precipitation seasonality (Table 2, Figure 2). No significant relationships between prevalence and either elevation, mean annual temperature, or annual precipitation were detected (Table 2).

**Table 2.** Spearman correlations between percentage of positive lizards and environmental predictors. Values of the correlation coefficient (rho) and its significance (p) are shown. Only the positive correlation with vegetation structure and negative correlation with precipitation seasonality were significant (cutoff level = 0.05)

Predictors	rho	p
Vegetation type	0.90	0.003
Elevation	0.29	0.251
Mean annual temperature	−0.31	0.231
Annual precipitation	0.19	0.332
Precipitation seasonality	−0.81	0.022

## Discussion

This study elaborates on the occurrence of *A. cantonensis* larvae in the lizard *G. galloti* as paratenic hosts on the Macaronesian island of Tenerife and demonstrates that this abundant lizard species can be used as a suitable sentinel for the presence of *A. cantonensis.* To reduce the possible impact on the population of endemic lizards, we used provoked caudal autotomy to obtain the biological material for the qPCR assay, and the lizards were immediately released.

In many isolated island systems, lizards are among the dominating, terrestrial vertebrate fauna, and lizard-based active surveillance could replace rat trapping, which can be highly demanding in terms of time and labour, especially in xeric vegetation types of arid localities. Samples from lizard tails could provide information about not only the parasite’s presence but also infection intensities, with easier access and with no need of sacrificing protected species. However, while this method is convenient and efficient for epidemiological surveys, it cannot fully replace the collection of adult nematodes from a particular area of interest, as it cannot provide as much DNA and thus may prove insufficient for more complex molecular and phylogenetic analyses. Traditionally, the surveillance of *A. cantonensis* has focused heavily on its occurrence in molluscs [29–31]. However, in contrast to reptiles, most of the gastropod species are generally active only in wet seasons, and thus they might not be easily accessible throughout the year, especially in xeric habitats. Also, on ecologically diverse islands such as Tenerife, it is not possible to collect the same species of snails or slugs throughout the island, which makes comparison among localities problematic.

The results of this study demonstrate that lizards can be useful for monitoring the distribution of *A. cantonensis.* Additionally, these results are the first to confirm the unequivocal presence of the parasite in the arid south of the island (using molecular tools). The parasite’s DNA was present in lizards from all localities except Erjos, which may be because of the small sample size (only three lizards were trapped there). Furthermore, Erjos is situated at higher elevations than the other localities, potentially influencing the presence of the parasite, although our available data are insufficient to confirm this hypothesis. There was no significant difference in the prevalence among adult males, adult females, and juveniles, although the small sample size of juveniles (only two positive individuals) precluded meaningful statistical comparison. Similarly, because we only had two positive juveniles, we compared infection intensities only between adult males (13 positive out of 38) and adult females (20 out of 81 positive), finding no significant difference.

The prevalence of *A. cantonensis* infection was positively correlated with vegetation cover. In all three localities with sparse vegetation cover (the most xeric habitats: Tejina, Valle San Lorenzo, Maria Jimenez), the percentages of positive lizards were the lowest (4.5–11.1%, Table 1). Conversely, in the single woodland locality (Anaga), the prevalence was the highest (64%), while sites in the intermediate vegetation categories 2, 3, and 4 exhibited intermediate prevalences. We made consistent efforts (the same time spent and number of traps used at each locality) to collect a sufficient number of lizards, aiming for a sample size that could be treated as representative of the population density. However, sample size varied among localities because capturing lizards in the more humid environments was more challenging [24] (only 11 lizards were collected from the woodland, with an average of 19 lizards per xeric locality, i.e., bare rocks with succulents). Therefore, effective collection strategies may vary in different habitats if the goal is to maximize lizard sample collection. The presence of gastropods and rats is one of the key factors determining the parasite’s occurrence. Gastropods usually thrive in environments with elevated humidity (which correlates positively with increasing vegetation cover) [31, 32]. Additionally, both rat species present in the Canary Islands, *Rattus rattus* and *Rattus norvegicus*, are more abundant in the presence of vegetation [33, 34]. Even in the xeric localities we examined, there was some limited vegetation near the trap locations. In the xeric southern part of Tenerife, the parasite seems to be able to find enough suitable hosts, perhaps not enough to thrive but sufficient for survival.

The prevalence of *A. cantonensis* in lizards was negatively correlated with precipitation seasonality, meaning that the greater the variation in rainfall throughout the year, the lower the percentage of infected lizards. Since the unevenness of rainfall negatively influences the abundance of gastropods and rats [35, 36], the lower *A. cantonensis* occurrence in the habitats with high rainfall seasonality is probably the result of less suitable conditions for the hosts. Moreover, nematodes native to tropical regions accustomed to consistent precipitation throughout the seasons may face challenges in adapting to more pronounced seasonality. Nevertheless, for an accurate prediction of the nematode distribution in Tenerife (and in similar ecosystems in locations other than the Canary Islands), additional studies incorporating a broader range of suitable hosts are necessary.

## Supporting information

Anettová et al. supplementary material Anettová et al. supplementary material

## Data Availability

All data generated or analyzed during this study are included in this published article and its supplementary information files. The dataset will be available with at http://doi.org/10.1017/S0950268824000931.

## Supplementary material

For supplementary material accompanying this paper visit http://doi.org/10.1017/S0950268824000931. click here to view supplementary material
