# Supplementary material for: Lizards as sentinels for the distribution of Angiostrongylus cantonensis
Source: Epidemiol Infect. 2024 Dec 13;152:e168. doi: 10.1017/S0950268824000931 (PMC11696602; doi:10.1017/S0950268824000931)
Supplement: Anettová et al. supplementary material [file S0950268824000931sup001.zip › Supplement Table S1.docx]

| Sample name | Locality |  | | | *A. cantonensis* DNA | Ct | Mean concentration | Stdeva concentration | No. of L3 per gram of tissue |
| --- | --- | --- | --- | --- | --- | --- | --- | --- | --- |
|  |  | **Nearest settlement** | **Elevation (m)** | **Vegetation type** |  |  |  |  |  |
| RK Liz 1 (F) | **Loc 1** | Tanque/Tierra del Trigo | 460 | 3 | **Positive** | 24.43 | 2.37E-03 | 2.33E-04 | 5.63E-02 |
| Tail Liz 2-3  (M, F) | **Loc 1** | Tanque/Tierra del Trigo | 460 | 3 | neg. |  |  |  |  |
| Tail Liz 4 (juv.) | **Loc 1** | Tanque/Tierra del Trigo | 460 | 3 | **Positive** | 29.46 | 1.09E-04 | 4.24E-06 | 7.96E-03 |
| Tail Liz 5 (M) | **Loc 2** | Garachico | 160 | 2 | **Positive** | 29.7 | 7.98E-04 | 5.87E-05 | 2.43E-02 |
| Tail Liz 6-8  (1x F, 2x M) | **Loc 2** | Garachico | 160 | 2 | neg. |  |  |  |  |
| Tail Liz 9 (F) | **Loc 2** | Garachico | 160 | 2 | **Positive** | 28.01 | 2.92E-04 | 3.89E-05 | 7.79E-03 |
| Tail Liz 10 (F) | **Loc 2** | Garachico | 160 | 2 | **Positive** | 23.09 | 5.76E-03 | 2.12E-04 | 1.79E-01 |
| Tail Liz 11-13 (F) | **Loc 2** | Garachico | 160 | 2 | neg. |  |  |  |  |
| Tail Liz 14 (F) | **Loc 2** | Garachico | 160 | 2 | **Positive** | 28.38 | 2.60E-04 | 7.07E-05 | 8.33E-03 |
| Tail Liz 15-16 (F) | **Loc 2** | Garachico | 160 | 2 | neg. |  |  |  |  |
| Tail Liz 17 (F) | **Loc 2** | Garachico | 160 | 2 | **Positive** | 23.57 | 3.87E-03 | 6.86E-04 | 1.04E-01 |
| Tail Liz 18-21 (2x juv, 2x F) | **Loc 2** | Garachico | 160 | 2 | neg. |  |  |  |  |
| Tail Liz 22 (M) | **Loc 3** | Erjos | 1180 | 4 | neg. |  |  |  |  |
| RK Liz 23 (F) | **Loc 1** | Tanque/Tierra del Trigo | 460 | 3 | neg. |  |  |  |  |
| Tail Liz 24-25 (juv) | **Loc 3** | Erjos | 1180 | 4 | neg. |  |  |  |  |
| Tail Liz 26 (M) | **Loc 4** | Tegueste | 370 | 4 | **Positive** | 34.42 | 6.92E-06 | 2.62E-06 | 1.64E-04 |
| Tail Liz 27-31 (F) | **Loc 4** | Tegueste | 370 | 4 | neg. |  |  |  |  |
| RK Liz 32 (juv.) | **Loc 4** | Tegueste | 370 | 4 | **Positive** | 33.64 | 8.01E-06 | 3.52E-06 | 1.85E-04 |
| Tail Liz 33 (M) | **Loc 5** | Anaga | 660 | 5 | **Positive** | 29.74 | 8.48E-05 | 5.87E-06 | 2.83E-03 |
| Tail Liz 34 (F) | **Loc 5** | Anaga | 660 | 5 | **Positive** | 30.88 | 3.63E-05 | 9.76E-06 | 1.07E-03 |
| Tail Liz 35 (F) | **Loc 5** | Anaga | 660 | 5 | **Positive** | 30.15 | 7.31E-05 | 6.29E-06 | 2.71E-03 |
| Tail Liz 36-42 (3x M, 4x F) | **Loc 6** | Tejina | 260 | 1 | neg. |  |  |  |  |
| Tail Liz 43 (M) | **Loc 6** | Tejina | 260 | 1 | **Positive** | 33 | 9.57E-06 | 2.74E-06 | 4.16E-04 |
| Tail Liz 44-51 (3x M, 5x F) | **Loc 6** | Tejina | 260 | 1 | neg. |  |  |  |  |
| Tail Liz 52 (M) | **Loc 7** | Valle San Lorenzo | 420 | 1 | neg. |  |  |  |  |
| Tail Liz 53 (F) | **Loc 7** | Valle San Lorenzo | 420 | 1 | **Positive** | 28.1 | 1.92E-04 | 3.25E-05 | 8.81E-03 |
| Tail Liz 54-55 (F, M) | **Loc 5** | Anaga | 660 | 5 | neg. |  |  |  |  |
| Tail Liz 56 (F) | **Loc 5** | Anaga | 660 | 5 | **Positive** | 30.01 | 6.33E-05 | 1.73E-05 | 4.90E-04 |
| Tail Liz 57 (F) | **Loc 5** | Anaga | 660 | 5 | **Positive** | 29.14 | 8.55E-05 | 6.15E-05 | 1.38E-03 |
| Tail Liz 58 (M) | **Loc 5** | Anaga | 660 | 5 | **Positive** | 32.74 | 1.29E-05 | 6.36E-07 | 1.96E-04 |
| Tail Liz 59 (M) | **Loc 5** | Anaga | 660 | 5 | **Positive** | 33 | 7.97E-06 | 1.97E-06 | 9.76E-05 |
| Tail Liz 60-61 (F) | **Loc 8** | María Jiménez | 70 | 1 | neg. |  |  |  |  |
| Tail Liz 62 (F) | **Loc 9** | El Rincon (La Orotava) | 190 | 3 | neg. |  |  |  |  |
| Tail Liz 63 (F) | **Loc 9** | El Rincon (La Orotava) | 190 | 3 | **Positive** | 30.99 | 3.44E-05 | 8.41E-06 | 1.07E-03 |
| Tail Liz 64 (M) | **Loc 9** | El Rincon (La Orotava) | 190 | 3 | **Positive** | 33.45 | 8.05E-06 | 4.67E-06 | 1.98E-04 |
| Tail Liz 65 (M) | **Loc 9** | El Rincon (La Orotava) | 190 | 3 | **Positive** | 33 | 9.80E-06 | 4.67E-06 | 2.08E-04 |
| Tail Liz 66-67 (M, juv) | **Loc 9** | El Rincon (La Orotava) | 190 | 3 | neg. |  |  |  |  |
| Tail Liz 68 (M) | **Loc 9** | El Rincon (La Orotava) | 190 | 3 | **Positive** | 30.29 | 7.62E-05 | 1.91E-05 | 3.20E-03 |
| Tail Liz 69 (F) | **Loc 9** | El Rincon (La Orotava) | 190 | 3 | **Positive** | 32.69 | 1.22E-05 | 2.33E-06 | 4.48E-04 |
| Tail Liz 70 (M) | **Loc 9** | El Rincon (La Orotava) | 190 | 3 | **Positive** | 30.99 | 4.10E-05 | 9.90E-07 | 1.01E-03 |
| Tail Liz 71 (F) | **Loc 9** | El Rincon (La Orotava) | 190 | 3 | **Positive** | 28.74 | 1.76E-04 | 1.41E-05 | 3.91E-03 |
| Tail Liz 72 (F) | **Loc 9** | El Rincon (La Orotava) | 190 | 3 | **Positive** | 29.65 | 8.51E-05 | 1.22E-05 | 3.65E-03 |
| Tail Liz 73 (M) | **Loc 9** | El Rincon (La Orotava) | 190 | 3 | **Positive** | 33.75 | 7.95E-06 | 4.98E-06 | 1.75E-04 |
| Tail Liz 74 (F) | **Loc 9** | El Rincon (La Orotava) | 190 | 3 | **Positive** | 32.96 | 1.13E-05 | 4.95E-07 | 2.42E-04 |
| Tail Liz 75 (M) | **Loc 9** | El Rincon (La Orotava) | 190 | 3 | **Positive** | 32.42 | 1.83E-05 | 2.62E-06 | 3.82E-04 |
| Tail Liz 76 (M) | **Loc 9** | El Rincon (La Orotava) | 190 | 3 | neg |  |  |  |  |
| Tail Liz 77 (M) | **Loc 9** | El Rincon (La Orotava) | 190 | 3 | **Positive** | 30.54 | 3.28E-04 | 6.36E-05 | 8.58E-06 |
| Tail Liz 78 (F) | **Loc 9** | El Rincon (La Orotava) | 190 | 3 | neg |  |  |  |  |
| Tail Liz 79 (F) | **Loc 9** | El Rincon (La Orotava) | 190 | 3 | **Positive** | 29.93 | 3.40E-04 | 7.50E-05 | 1.20E-05 |
| Tail Liz 80-85 (3x F, 2x M, 1x juv) | **Loc 9** | El Rincon (La Orotava) | 190 | 3 | neg |  |  |  |  |
| Tail Liz 86-87 (F, juv) | **Loc 5** | Anaga | 660 | 5 | neg |  |  |  |  |
| Tail Liz 88 (F) | **Loc 4** | Tegueste | 370 | 4 | **Positive** | 27.51 | 1.50E-03 | 2.83E-05 | 5.29E-05 |
| Tail Liz 89 (F) | **Loc 4** | Tegueste | 370 | 4 | **Positive** | 28.83 | 6.95E-04 | 3.75E-05 | 2.27E-05 |
| Tail Liz 90-91 (M, juv) | **Loc 4** | Tegueste | 370 | 4 | neg |  |  |  |  |
| Tail Liz 92 (F) | **Loc 4** | María Jimenez | 70 | 1 | **Positive** | 28.68 | 8.06E-04 | 3.04E-05 | 2.52E-05 |
| Tail Liz 93-94 (F) | **Loc 4** | María Jiménez | 70 | 1 | neg |  |  |  |  |
| Tail Liz 95-114 (14x F, 6x M) | **Loc 7** | Valle San Lorenzo | 420 | 1 | neg |  |  |  |  |
| Tail Liz 115-128 (12x F, 2x M) | **Loc 8** | María Jiménez | 70 | 1 | neg |  |  |  |  |
| Tail Liz 129 (M) | **Loc 8** | María Jiménez | 70 | 1 | **Positive** | 31.4 | 2.53E-04 | 4.95E-05 | 9.44E-03 |

**Supplementary Table S1:** Quantitative qPCR analysis was performed on *Angiostrongylus cantonensis* DNA extracted from the tails of *Gallotia galloti*. The resulting data were utilized for statistical comparisons of larval DNA quantities between males (M) and females (F). Each analysis was conducted in duplicate, and the average value (Mean concentration) was used to determine the estimated amount of *A. cantonensis* L3 per gram of tissue. Stdeva concentration represents the standard deviation between the duplicate concentrations.
