# Supplementary material for: Lizards as sentinels for the distribution of Angiostrongylus cantonensis
Source: Epidemiol Infect. 2024 Dec 13;152:e168. doi: 10.1017/S0950268824000931 (PMC11696602; doi:10.1017/S0950268824000931)
Supplement: Anettová et al. supplementary material [file S0950268824000931sup001.zip › Supplementary Table S2.docx]

| **Name** | **Ct** | **Concentration** |
| --- | --- | --- |
| 1L3 dil.1000x | 27.07 | 9.93E-04 |
| 1L3 dil.1000x | 27.05 | 1.01E-03 |
| 1L3 dil.100x | 23.19 | 1.02E-02 |
| 1L3 dil.100x | 23.26 | 9.75E-03 |
| 1L3 dil.10x | 19.64 | 9.93E-02 |
| 1L3 dil.10x | 19.62 | 1.01E-01 |
| 1L3 | 16.01 | 1.10E+00 |
| 1L3 | 16.28 | 9.12E-01 |

**Supplementary Table S2:** Ct values and concentration resulting from qPCR analysis setting the standard curve calculated using serial dilutions (1x, 10x, 100x and 1000x) of DNA extracted from a single L3 of *Angiostrongylus cantonensis*.
