# Supplementary figures and images for: Lizards as sentinels for the distribution of Angiostrongylus cantonensis
Source: Epidemiol Infect. 2024 Dec 13;152:e168. doi: 10.1017/S0950268824000931 (PMC11696602; doi:10.1017/S0950268824000931)

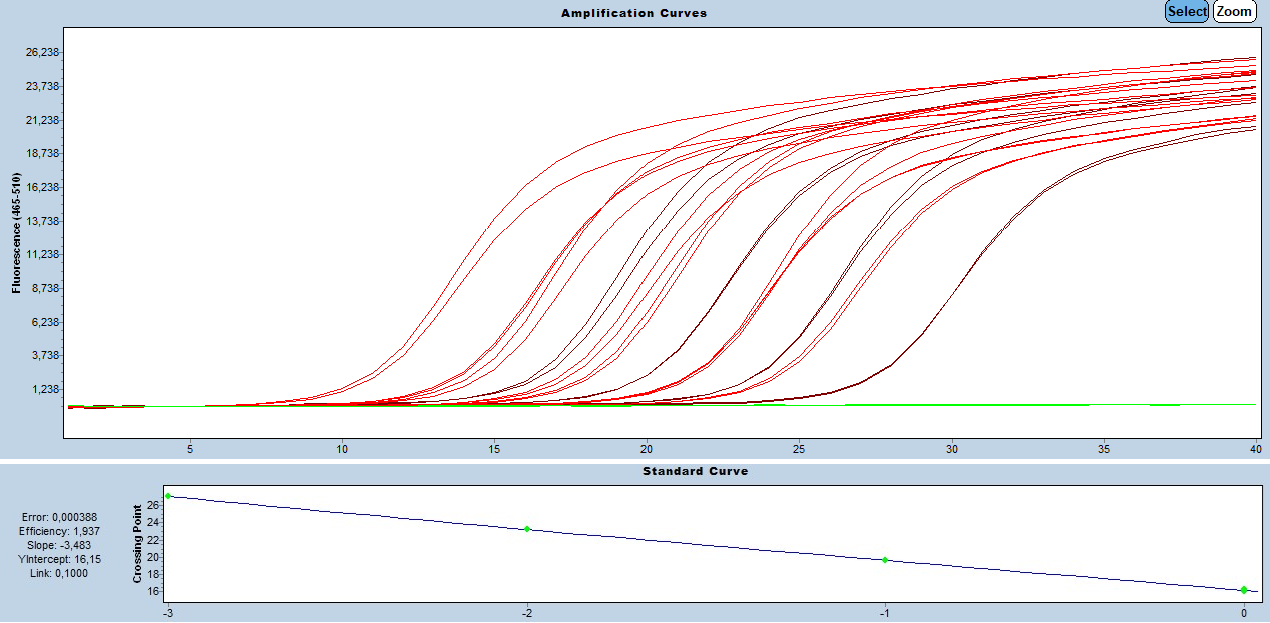

Supplement: Anettová et al. supplementary material [file S0950268824000931sup001.zip › Supplementary_Figure_F1_standard_curve.png]
